# Epitope Specificity of Anti-Citrullinated Protein Antibodies

**DOI:** 10.3390/antib6010005

**Published:** 2017-03-08

**Authors:** Nicole H. Trier, Gunnar Houen

**Affiliations:** Department of Autoimmunology and Biomarkers, Statens Serum Institut, Artillerivej 5, 2300 Copenhagen S, Denmark

**Keywords:** anti-citrullinated protein antibodies, citrullinated epitopes, rheumatoid arthritis, cyclic citrullinated peptides

## Abstract

Anti-citrullinated protein antibodies are primarily associated with a progressive course in the autoimmune disease rheumatoid arthritis, a disease with a chronic and inflammatory nature. These antibodies do not appear to have any strict dependency for reactivity except from the presence of the non-genetically encoded amino acid citrulline, which is the result of a posttranslational modification, catalyzed by calcium-dependent peptidylarginine deiminase enzymes. Nevertheless, several amino acids surrounding the citrulline residue notably influence antibody reactivity, especially with a central-Cit-Gly-motif being essential for antibody reactivity. Most importantly, these antibodies have been proposed to be divided into two groups, based on their ability to recognize multiple citrullinated peptides. Thus, an “overlapping” antibody group, which appears to recognize several citrullinated peptides, and a “non-overlapping” antibody group, which only recognizes a limited number of citrullinated peptides, have been proposed. Based on these findings, we suggest that antibodies recognizing several citrullinated targets, also referred to as cross-reactive antibodies, primarily are backbone-dependent, whereas less cross-reactive antibodies primarily depend on the side chains of the amino acids comprising the epitopes for stable antibody-antigen interactions, which reduces the degree of cross-reactivity significantly. Clarifying the reactivity pattern of anti-citrullinated protein antibodies may contribute to determining their true nature of origin.

## 1. Introduction to Anti-Citrullinated Protein Antibodies

### 1.1. Citrullination of Proteins by Peptidylarginine Deiminases

**Figure 1:**
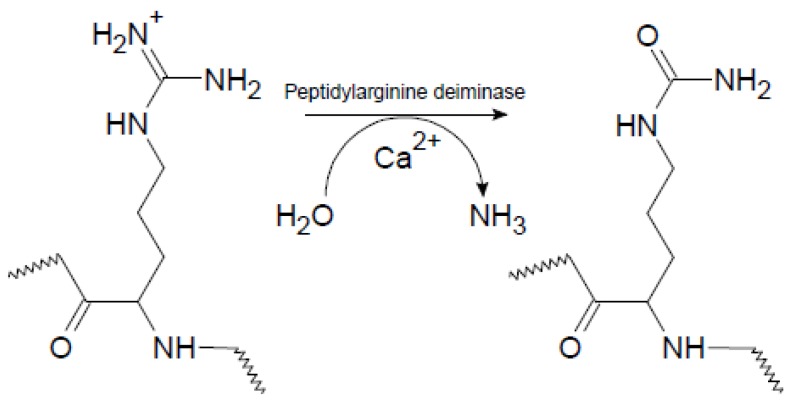
Citrullination (deimination) of peptidylarginine catalyzed by peptidylarginine deiminase in a calcium-dependent process. The positively charged guanidino group is replaced with the neutral ureido group.

Rheumatoid arthritis (RA) is a systemic autoimmune disease characterized by chronic inflammation in the synovial joints, which ultimately may lead to joint destruction and erosion of the underlying bone [1]. One of the most important serological discoveries in rheumatology has been the identification of autoantigens in RA, containing epitopes with the non-genetically encoded amino acid citrulline (Cit) [2,3]. Cit is a post-translationally modified, deiminated derivative of Arg, which originally was named after the watermelon *Citrullus vulgaris*, which contains a large amount of free Cit. The process of citrullination, or deimination, involves the enzymatic conversion of Arg-containing proteins, which usually occurs during inflammatory processes [4]. Citrullination is catalyzed by peptidylarginine deiminase enzymes (PADs), which are calcium-dependent metalloenzymes, belonging to the superfamily of guanidino group–modifying enzymes [4]. Under physiological conditions, PAD enzymes are normally inactive due to low concentrations of intracellular calcium. Once activated, as a result of elevated calcium levels, the PAD enzymes citrullinate a range of cytoplasmic, nuclear, membrane and mitochondrial proteins [5]. In this process, the positively charged guanidino group of Arg is replaced with the neutral ureido group, as depicted in Figure 1. The result of this conversion is a very small change in molecular mass and the loss of a positive charge. This apparently “small” change in the amino acid side chain has a profound effect on intramolecular and intermolecular interactions, as ionic and hydrogen bond interactions are influenced, which changes the isoelectric potential of the proteins leading to structural unfolding, thereby causing more open configurations for the proteins and proteolytic degradation [6].

Citrullination of proteins normally occurs in cells undergoing apoptosis, where citrullinated proteins are cleared from the body and not encountered by the immune system. In this process, unlimited influx of extracellular calcium activates PAD enzymes, as calcium levels become higher than the resting physiological concentration [7]. Citrullination marks intracellular proteins for degradation with a complete loss both of the polymerization competence of the intermediate filament proteins and of the filament-forming ability [8]. Following citrullination, apoptotic bodies are engulfed by phagocytes, thereby preventing inflammatory reactions. However, in RA, citrullinated proteins are not effectively cleared. In fact, a dysregulation of apoptosis eventually in combination with the ineffective clearance of apoptotic material is suspected to be involved in the accumulation of dying cells and the exposition of autoantigens in RA [9]. Consequently, citrullinated proteins are exposed to the immune system, leading to the generation of citrulline-specific antibodies [10]. This is supported by studies describing that the impaired apoptosis of RA fibroblast-like synoviocytes is pivotal in the establishment of the inflammatory process [11].

In this process, an increase in PAD2 and PAD4 enzyme levels is often observed [7,12]. The role of PAD in the onset of RA has been examined, and recently, a model for PAD infiltration into the synovial joints of RA patients was presented, where the PAD2 and PAD4 enzymes, expressed in monocytes and macrophages, are recruited to the joints [13]. These studies described that the activated cells, containing unstimulated PADs, eventually undergo apoptosis where the PADs are activated as a result of elevated calcium levels. Activation of PAD results in the citrullination of proteins, after which PAD enzymes diffuse out of the cell to citrullinate extracellular proteins. For example, PAD2 and PAD4 enzymes have been reported to citrullinate fibrin in the affected joints. Nevertheless, antibodies to citrullinated proteins have been reported to be present in the sera several years before the clinical symptoms of RA appear [14,15]; hence, the dysregulated PAD activity is potentially disease-initiating. It is currently not known what causes PADs to become dysregulated, but several scenarios may lead to abnormal levels of citrullinated proteins. e.g., PAD activity may become uncontrolled at high calcium concentrations and may lose target specificity, which would result in the extensive modification of arginine-rich proteins and ultimately the loss of activity [7]. Increased levels of PAD enzymes could also explain the elevated levels of citrullinated proteins; however, it is unknown what might cause increased translation [7,12]. Moreover, it has been proposed that calreticulin interacts with components of the immune system and promotes immune dysregulation [16,17,18]. Calreticulin is essential for the clearance of dying cells and has been reported to act as a signal-transducing receptor for the shared epitope (SE) found in RA-associated major histocompatibility complex (MHC) class II molecules [19].

### 1.2. Physiological Functions of Citrullination

Although typically occurring under inflammatory conditions, citrullination has several physiological roles, e.g., citrullination has a role in brain development, female fertility, hair formation and regulation of gene expression via chromatin remodeling, as presented in Table 1 [20,21,22,23]. Citrullination of p53 and the estrogen receptor is essential for the regulation of these proteins in the p53- and estrogen-associated pathways [24,25,26,27,28,29,30]. Moreover, citrullination is involved in skin keratinization and several immune functions [7,31,32,33,34,35,36]. In skin keratinization, citrullination of keratin alters the protein structure, enabling proteins to bind to it, which ultimately results in the generation of a protective matrix in the skin [7]. In addition, citrullination of myelin basic protein is important in the function of the myelin sheath, as well as in the plasticity of the central nervous system [7]. In immune functions, citrullination is involved in the function of chemokines and cytokines and it participates in antibacterial neutrophil extracellular trap (NET) formation [37].

As presented in Table 1, the process of citrullination is not specific to RA; however, an aberrant B cell response against citrullinated epitopes may be specific to RA. Moreover, other rheumatologic diseases with synovitis, including inflammatory osteoarthritis, reactive arthritis, undifferentiated arthritis, gout and even trauma, and in rare cases healthy individuals as well, are occasionally associated with the presence of antibodies to citrullinated proteins [38,39,40]. Nevertheless, these antibodies are detected in a very small percentage of cases as compared to RA; thus, antibodies to citrullinated proteins are specific diagnostic and prognostic markers in RA [41].

### 1.3. Characteristics of Anti-Citrullinated Protein Antibodies

Antibodies recognizing the post-translationally modified amino acid Cit are referred to as anti-citrullinated protein antibodies (ACPA). These antibodies are primarily directed to citrullinated proteins located in the joints or present in circulation [42,43,44]. Moreover, recent studies have reported that ACPA occasionally are enriched in the lung as well, early in the development of RA [45].

ACPAs belonging to different immunoglobulin classes have been identified [46,47]. Although ACPAs predominantly are of the IgG class, IgM and IgA classes are present as well. Consequently, several commercial assays are available, which primarily detect the presence of ACPA IgG, whereas one assay detects the presence of IgA and IgG, reportedly yielding a higher assay sensitivity. Although the ACPA IgM antibodies especially are short-lived, IgM reactivity responses have been characterized [48]. Interestingly, the antibody isotype appears to influence the reactivity of ACPA responses, as it has been reported that ACPA IgM responses are different from ACPA IgG responses [48]. Similarly, the ACPA response against one citrullinated antigen is different from the immune response against another citrullinated protein. Elucidation of the mechanisms behind these observations could be of relevance to the identification of the true citrullinated antigens that drive ACPA responses [48].

The presence of ACPAs is associated with poor disease outcomes, such as increased disease activity, radiographic progression, disability and increased mortality [49,50,51,52]. In addition, studies find that the presence of ACPA in patients with early arthritis predicts disease progression, as ACPA-positive RA is more severe and progressive than ACPA-seronegative RA [52,53,54].

The search for autoantigens associated with RA disease pathogenesis and the diagnosis of RA has resulted in the characterization of several citrullinated proteins and the development of several assays for detection of ACPA, which is a major breakthrough in the laboratory diagnostics of RA [55]. As a result, several types of ACPAs have been identified, as seen in Table 2. An ACPA originally reported in 1964 was the anti-perinuclear factor antibody [56]. Another ACPA, the anti-keratin antibody, was discovered in 1979 in RA sera [57]. Both antibodies recognize the cyto-keratin filament-aggregating protein filaggrin [58,59,60]. Nevertheless, as epidermal filaggrin is not expressed in the joints, it cannot be considered as a joint-specific autoantigen that drives an anti-filaggrin response. Thus, the antibody response should be the result of a cross-reaction against unknown proteins expressed in the joint. Another highly specific autoantibody discovered in 1994 was the anti-Savoie (anti-Sa) antibody [61,62]. The reactivity of Sa antibodies to citrullinated vimentin, defining them as ACPAs, was recently demonstrated [13]. Finally, a mutated and citrullinated version of vimentin has been used in a recently developed enzyme-linked immunosorbent assay (ELISA) for detection of anti-mutated citrullinated vimentin antibodies, anti-MCV [63]. Cyclic citrullinated peptide (CCP) antibodies were reported in 2000, and were named based on the reactivity in an ELISA using synthetic CCPs [64]. The sensitivity of the first generation anti-CCP test was further increased with the development of the CCP2, CCP3 and CCP3.1 tests [65,66,67,68]. Dependent on the type of antibody, varying levels of sensitivity and specificity are found in these assays, as shown in Table 2.

ACPAs are found in approximately 70% of RA cases and can be detected up to 14 years before the onset of disease [14,15,80,81]. Consequently, ACPAs were included in the 2010 American College of Rheumatology/European League Against Rheumatism classification criteria for RA [82], in contrast to the previous 1987 criteria, where the rheumatoid factor was the sole serological marker [83].

Routine testing for ACPAs is commonly performed by using a commercial anti-CCP2 ELISA. However, where the CCP1 assay is based on a filaggrin-derived peptide, the CCPs used in the CCP2 assay reportedly do not correspond to any human protein sequence, and thus the CCP2 antibodies only act as surrogate markers for autoimmunity in RA without formally defining any reactivity against autoantigens present in vivo.

Despite up to 70% of RA sera being estimated to be ACPA-positive, the absolute concentration of ACPA in serum currently remains unknown. The CCP2 assay, which currently is regarded as the golden standard assay, occasionally determines RA sera as ACPA-negative; however, it has been estimated that approximately 5%–8% of these sera in fact are ACPA-positive [79,84,85,86].

### 1.4. Pathogenesis of Rheumatoid Arthritis

In general, the pathogenesis of RA involves a number of cellular responses. Autoimmune responses mediated by T and B cells are important in the initiation of the inflammatory cascade. Then T cells, B cells, macrophages and neutrophils migrate into synovial tissues, where they, among others, produce proteases that destroy the extracellular matrix, in particular that of cartilage. As a result, synovial hypertrophy and angiogenesis occur, leading to cartilage destruction as well as osteoclast activation and ultimately bone erosion [87].

It has been a matter of much debate whether ACPAs play a role in the pathogenesis of RA, although the etiology of the disease remains unknown [88,89,90]. Using animal models it has been found that transfer of monoclonal antibodies recognizing citrullinated collagen or fibrinogen exacerbate inflammatory arthritis [90]. Moreover, ACPAs have been reported to be able to activate both FcR-positive cells and the complement system [91,92]. In addition, ACPAs are associated with a progressive disease course and a poor outcome [49,50,51,52]. Finally, the efficacy of (memory) B cell depletion in treating RA patients using a monoclonal anti-CD20 antibody (Rituximab) and the effect of plasmapheresis (in combination with TNF-α inhibitors and disease-modifying anti-rheumatic drugs (DMARDS) may suggest a pathogenic role of ACPAs [88,89,93,94]. Collectively, these findings argue that ACPAs could play a role in RA disease pathogenesis. Nevertheless, the long presence of ACPAs prior to the onset of the disease may indicate that the antibodies are not involved in the actual pathogenesis. Rather it has been proposed that ACPAs are associated with inflammation in the joints, as ACPAs in the inflamed synovium have been shown to be associated with citrullinated antigens forming immune complexes, resulting in progression of the inflammatory process [95]. These findings suggest that there is no target injury in the absence of citrullinated target antigens, but when these antigens are present, ACPAs can greatly amplify inflammation and damage. These findings are in accordance with the observation that ACPA-negative RA usually experiences a mild disease course, whereas ACPA-positive RA often experiences progressive disease and poor outcomes [49,50,51,52]. Finally, these findings are supported by an ex vivo study reporting that ACPA-containing immune complexes stimulate pro-inflammatory cytokine production in macrophages [91].

Only limited information is available in relation to citrulline-specific T cells in RA [96]. RA is thought to be a T cell–mediated disease, based on its strong association with human leukocyte antigen (HLA) class II alleles, clinical responsiveness to T cell–directed therapies and the presence of T cells in the synovium of early RA patients and in multiple joints [96,97,98]. Previous studies have demonstrated enhanced binding and presentation of citrullinated peptides by shared epitope (SE) HLA-DR and enhanced in vitro T cell responses directed against individual citrullinated epitopes [99,100,101,102,103]. Recent findings indicate that citrulline-specific Th1 cells are enriched in RA patients [96] and that T cells recognize citrullinated epitopes derived from multiple synovial antigens [103,104,105].

### 1.5. Rheumatoid Arthritis Risk Factors

As mentioned previously, the etiology of RA remains to be determined. Based on twin studies it has been proposed that the relative contribution of genetic variation to develop RA is 50%–60% [106,107]. The strongest evidence for the influence of genetic factors on RA onset relates to MHC class II antigens, and, in particular, to various HLA alleles, the most important of which is the MHC II structure defined by the SE [108]. In fact, it has been estimated that up to 50% of RA patients are SE-positive [105]. Stastny originally documented an association between HLA-DR4 (HLA-DRB1*04) and the risk of developing RA [108]. A discrepancy in the association of different HLA-DRB1 genes revealed the presence of a conserved hexapeptide sequence in the third hypervariable region of all RA-associated HLA-DRB1 alleles, involving positions 69–74 “EQK/RRAA”, also referred to as the SE [108]. The mechanism underlying the SE-RA association remains unclear; a common hypothesis attributes it to the presentation of arthritogenic antigens or T cell repertoire selection [109,110,111]. Moreover, studies by Kapitany and Snir found a significant correlation between HLA-DR4 and HLA-DRB1 and the ACPA titers, respectively [112,113]; hence it has been concluded that HLA-DR4 and HLA-DRB1 allele positivity poses an increased risk for developing RA because of their association with high ACPA titers.

Another genetic factor that is associated with increased risk of RA is the protein tyrosine phosphatase non-receptor type 22 (PTPN22). The PTPN22 gene encodes a tyrosine phosphatase, which is essential in T cell signaling [114,115].

In general, the currently known genetic risk factors associated with RA are thought to be specifically associated with either ACPA-positive or ACPA-negative disease. Thus, ACPA-positive RA has been found to be closely linked to the presence of HLA-DRB1 alleles containing SE motifs [116,117] and polymorphisms in the PTPN22 gene [115,118,119]. Moreover, ACPA-positive status has been suggested to be associated with the recently identified, but modest genetic risk factor TRAF1-C5 [120]. Other genetic factors such as variations in the interferon-regulating factor IRF-5 and polymorphisms in a newly identified risk gene in the C-type lectin complex have been suggested to be associated with ACPA-negative RA disease [121,122].

Further findings by Klareskog and colleagues showed that in HLA-DR–positive patients, cigarette smoking triggers the formation of ACPAs [116]. More recently, it has been shown that HLA-DRB1 SE, PTPN22 polymorphism and cigarette smoking together associate more significantly with ACPA reactivities than with anti-CCP levels, indicating that genetic as well as environmental factors are essential and interact with each other. Based on these findings, it has been proposed that further genetic analysis will allow for the separation of RA into subgroups, where each has specific risk factors and disease course [85,123].

## 2. Citrullinated Epitope Characteristics

Examples of reported citrullinated proteins associated with RA are, among others, fibrin, fibrinogen, enolase, collagen, vimentin and EBNA [124,125,126,127,128,129]. Currently, no dominant epitope has been identified among the citrullinated proteins, although it is evident that the number of citrullinated epitopes is limited initially, but increases over time. Once the disease has been diagnosed, the number of recognized epitopes remains stable [130]. This process of epitope spreading occurs prior to the onset of RA [80,130].

It is well established that ACPAs are directed to various citrullinated epitopes, in which not only Cit but also flanking amino acids are essential for antibody recognition [3,131,132]. Nevertheless, thorough analysis of citrullinated epitopes reveals no conclusive epitope pattern, and thus several citrullinated epitopes have been identified, as presented in Table 3.

**Figure 2:**
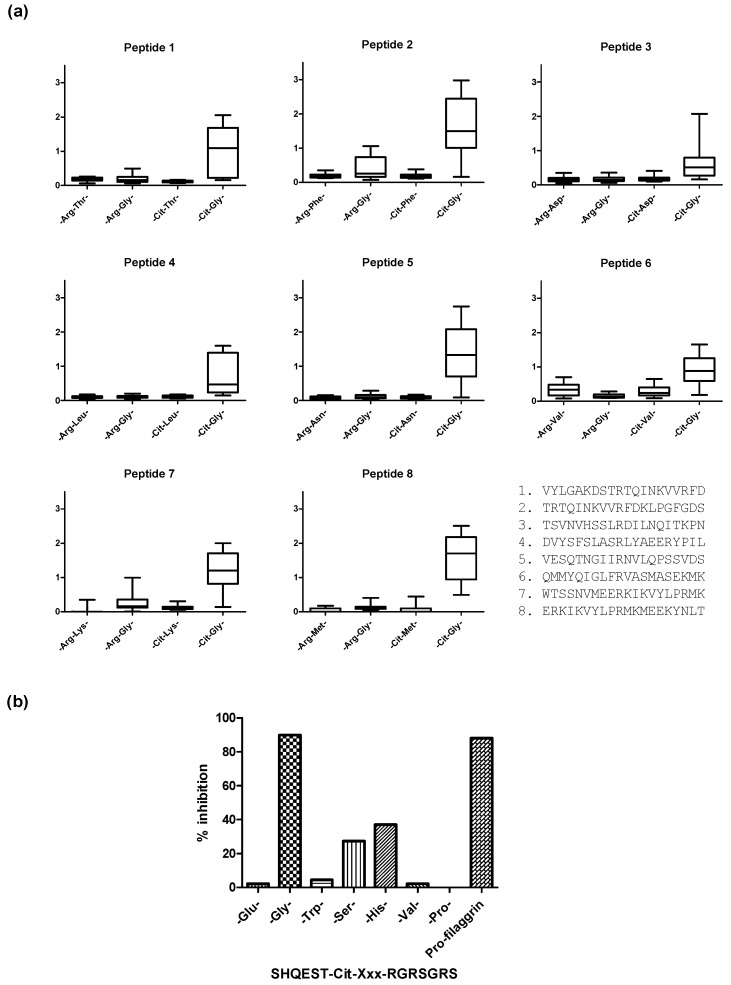
Anti-citrullinated protein antibody reactivity to substituted citrullinated peptides. (**a**) ACPA reactivity to substituted 20-mer ovalbumin peptides. Original peptides containing Arg in a central position were used as templates. In the remaining peptides, the Arg-Xxx motif was substituted with Arg-Gly, Cit-Xxx and Cit-Gly; (**b**) ACPA reactivity to substituted pro-filaggrin peptides. The peptide SHQEST-Cit-GRSRGRS was used as template. In the position C-terminal to Cit, various amino acid residues were introduced (Glu, Gly, Trp, Ser, His, Val, Pro). The 22-mer pro-filaggrin peptide (HQSHQEST-Cit-GRSRGRSGRSGS) was used as control.

Original findings analyzing ACPA reactivity described that citrullinated epitopes primarily depend on a combination of Cit with small neutral amino acid residues such as Ser or Gly, often in combination with Val, His or Thr [2,3,128,132,133,134]. Recent findings do, however, indicate that primarily the -Cit-Gly- motif is essential for antibody reactivity, as illustrated in Figure 2. Using 20-mer ovalbumin peptides containing Arg-Xxx residues in the center, the following substitutions of -Arg-Gly-, Cit-Xxx-, and -Cit-Gly- were introduced to examine the effect of the Cit-Gly motif. As seen in Figure 2a, only the peptides containing the Cit-Gly motif obtained significant antibody reactivity [131]. These findings were confirmed by Ala scanning studies, where antibody reactivity to substituted pro-filaggrin peptides was analyzed. Using a 14-mer citrullinated pro-filaggrin peptide as a template, we found that all amino acids could be substituted with Ala without reducing antibody reactivity, except from the central Cit-Gly motif [133,135]. Moreover, we found that antibody reactivity was significantly reduced when substituting Gly in the Cit-Gly motif with other amino acid residues such as Ser, Thr, His, Pro, Trp and Val, as illustrated in Figure 2b [131]. Furthermore, it has been reported that the N-terminal position next to Cit and the second and third position C-terminal to Cit are irrelevant for antibody reactivity, as several amino acids are tolerated in these positions [131]. In addition to a Cit-Gly motif, the presence of positively charged amino acids C-terminal to Cit does appear to stimulate interaction, as peptides containing a Cit-Gly motif in combination with a positively charged residue in the C-terminal yield higher sensitivities [44,84,135,136]. Thorough characterization of a Fab-collagen peptide complex (A-Cit-GLTGRPGDA) performed by Uysal and colleagues confirmed that Cit and a C-terminal Arg are essential for reactivity, as the side chains of these residues are involved in most of the interactions with the antibody [44].

Although the immediate dependency relates to the presence of the central Cit-Gly motif, recent findings indicate that the presence of Pro C-terminal to Cit influences ACPA reactivity negatively, as the presence of Pro in the first, second or third position C-terminal to Cit notably reduces ACPA reactivity, possibly by contributing to a rigid structural conformation [131,133,135].

Although primarily dependent on the citrullinated Cit-Gly motif, this dipeptide is not sufficient for antibody reactivity [133]. Screening the reactivity of antibodies to citrullinated peptides found that at least five amino acids should be present in the citrullinated peptide for it to be recognized [3,6,137]. These findings were confirmed by Uysal et al., where analyses of a Fab-peptide crystal structure showed that the Cit residue and seven flanking amino acid residues were essential for reactivity [44]. Further analysis indicates that for optimal ACPA reactivity, the citrullinated peptides should be at least 14 amino acids long [3,133,135].

Several studies have been conducted using multiple citrullinated peptides [128,132,138]. Thus, as presented in Table 3, some ACPA epitopes contain more than one citrullyl residue. Nevertheless, several studies indicate that these residues do not contribute equally to antibody reactivity [3,72,127,132]. Often a single residue predominantly contributes to the ACPA reactivity, whereas the remaining residues contribute weakly or not at all.

## 3. Citrullinated Epitope Structures

Only limited information is available on the specific interaction between ACPAs and citrullinated epitopes. It has previously been reported that citrullination of Arg residues by PAD enzymes depends on the conformation of the target protein, which could indicate that the peptide fold may affect the citrullination of the specific Arg residue and ultimately the structure of the citrullinated peptide [139]. Thus, one possibility is that citrullination of proteins mediates distortion, initiating the exposure of neo-epitopes. Another possibility is that removal of the imine group from the Arg side chain may introduce unique specific molecular interactions, which may influence the protein structure [44]. This is in accordance to a theory proposed in 2009, which states that partial unfolding/denaturation by citrullination of particular Arg residues could initiate a mechanism which induces conformational changes of the peptide, resulting in the generation of β-hairpin structures [44]. However, unless the side chain of Arg directly contributes to an intramolecular ionic interaction, it appears most likely that citrullination results in exposure of neo-epitopes rather than inducing molecular changes.

Early findings hypothesized that since no sequence homology was evident between the citrullinated epitopes, structural homology should be of importance to ACPA antibodies [128]. These findings were primarily based on cross-reactivity analyses between a fibrinogen peptide and a filaggrin-derived peptide antibody [128]. Nevertheless, as no specific residues or groups of residues are favored in specific positions, it seems unlikely that these epitopes are located in specific structures. This is in accordance to recent findings, describing that ACPA antigenicity virtually is obtained with any peptide when introducing a Cit-Gly motif, and that ACPA reactivity primarily depends on the presence of a Cit-Gly motif in combination with a peptide backbone for stable reactivity, rather than a specific secondary structure [131,135]. Thus, in order for ACPAs to recognize multiple peptides, in theory, independent of amino acid composition and epitope structure, the peptides are believed to be of flexible nature [133,135].

It has been widely examined whether cyclic or linear epitope structures influence antibody reactivity [64,84,133,140]. In the original study by Schellekens et al. (2000), describing the reactivity to the first-generation CCP assay, synthetic CCPs were employed, which obtained higher sensitivity compared to linear versions [64]. Cyclic peptides were used in an attempt to force the peptide to adopt a β-hairpin conformation, as Cys-bridged peptides have been shown to mimic the β-turn structure of the antigenic epitope and can bind with enhanced affinity to antibodies [64,140,141]. Adoption of a β-hairpin structure is energetically more stable, and hence it has been proposed that the energetically favored peptide conformation may contribute to the predominance of β-turns compared with other possible structures in peptide-antibody complexes [142]. In relation to this, cyclization of citrullinated peptides has been reported to yield a more stable epitope presentation, which has been verified when conducting competitive inhibition experiments using cyclic and linear peptides [84]. Although cyclic peptides at first glance appear to yield higher ACPA sensitivities [64,137], recent findings indicate that reducing the number of amino acids in the cyclic structure has a profound negative influence on ACPA reactivity [133,137], most likely because the peptides are constrained in a rigid conformation, reducing the flexibility of the peptide [133,136].

**Figure 3:**
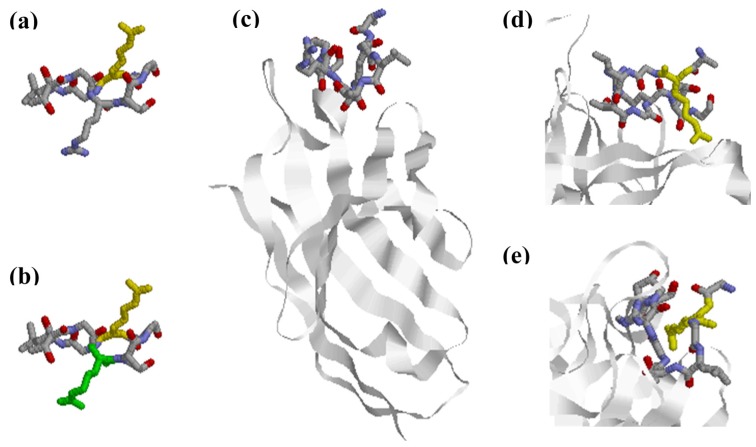
Anti-citrullinated collagen type II antibody in complex with citrullinated collagen II peptide aa 359–369. (**a**) Location of Cit (yellow) in the selected epitope; (**b**) Location of Cit (yellow) and Arg (green) extending in opposite directions; (**c**) Location of the citrullinated epitope in relation to the antigen-binding site of the antibody; (**d**) Location of Cit in the collagen II peptide relative to the antibody-binding groove; (**e**) Orientation of Cit into the antibody-binding groove. PDB ID: 2W65 [44].

Thorough studies performed by Uysal and colleagues characterized the interaction between a panel of mouse monoclonal antibodies generated and a triple helical collagen epitope (aa 359–369; A-Cit-GLTGRPGDA) [44]. Structure analysis of a monoclonal Fab fragment in complex with the citrullinated epitope showed that the citrullinated epitope changes conformation upon interaction with the citrulline-dependent antibody [44] by adopting a non-native β-hairpin conformation, where the side chain of Cit is exposed into the combining site of the antibody, as shown in Figure 3. This allows the peptide to have more interactions between the antibody’s complementarity-determining region (CDR) loops and the side chains of the amino acids, which contributes to an increased affinity. Moreover, side chains of the Arg and Cit residues are extended outwards in opposite directions, which increases the surface area of the interaction between the antibody and the epitope, as seen in Figure 3. The contact with the Cit residue indicates that an Arg in the same position would not have fitted in [44]. Based on these findings, it was concluded that Cit specificity can be explained by direct interactions, although it was not excluded that citrullination additionally changed the conformational structure of the targeted protein, which may lead to exposure of newly generated neo-epitopes.

Collectively, these findings indicate that peptides of flexible nature, eventually possible of folding into a β-turn structure, are essential for ACPA reactivity [44,135].

## 4. Anti-Citrullinated Protein Antibody Specificities

The facts that several citrullinated antigens have been identified in RA and that no immediate homology exists indicate that ACPA responses are cross-reactive, either by being positive for antibodies with different specificities or single antibodies that are true cross-reactors. Moreover, the golden standard for ACPA detection, the anti-CCP2 ELISA, does not contain epitopes from citrullinated proteins found in the joints, yet still obtains high antibody reactivity, which supports that these antibody responses indeed are cross-reactive. Thus, although RA sera may have high CCP antibody titers, does this not provide information about the identity of citrullinated proteins, which are recognized by the CCP antibodies.

**Figure 4:**
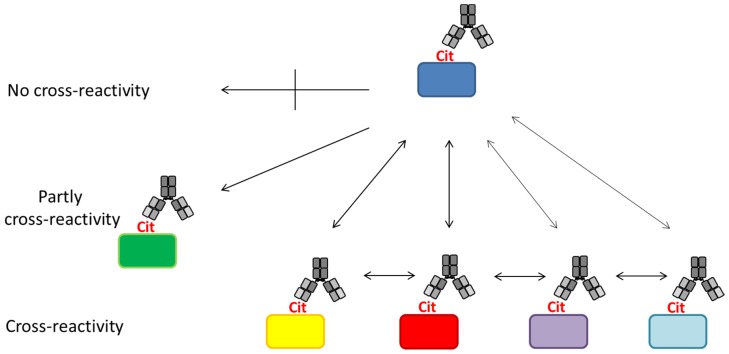
Anti-citrullinated protein antibodies are a collection of partially cross-reactive antibodies recognizing citrullinated targets. ACPA reactivities can be divided into several (partially) cross-reactive fine specificities. Some ACPAs captured by a citrullinated protein cross-react with other citrullinated proteins. A small group of ACPAs appears to have limited or no cross-reactivities. Different colors indicate different proteins.

The principle of ACPA reactivity is depicted in Figure 4. As seen, ACPAs have been reported to recognize a diverse set of citrullinated proteins [112,143,144]. However, a small group of ACPAs appear to be less cross-reactive or even epitope-specific; thus, they only recognize a single citrullinated epitope [79,143,144]. This makes ACPAs a collection of (partially) cross-reactive antibodies.

The degree of cross-reactivity has been analyzed in several studies. In a study by Montes and colleagues, the association between anti-citrullinated vimentin, anti-citrullinated α-enolase and CCP2-positivity was determined [145]. Interestingly, only a limited number of sera were found to recognize both peptides. Nevertheless, as the vimentin peptide (aa 60–75) does not contain any Cit-Gly motifs, which have been reported to be essential for reactivity [131], the low cross-reactivity is ascribed to the absence of the motif. Despite this association, it is unclear whether the antibodies recognizing the CCP2 peptides are the same antibodies that recognize citrullinated proteins in the joints. This is a drawback of several studies analyzing ACPA reactivity, as these studies often are based on CCP2-positive sera. In these cases, CCP2-negative RA sera, which may be ACPA-positive, are neglected.

Previous studies have indicated that ACPA reactivity patterns to citrullinated antigens, at the population level, vary between RA individuals [43,79,112,144]. In relation to this, different subgroups of ACPA antibodies have been determined, indicating that ACPA responses are highly diverse with respect to the recognition of specific citrullinated epitopes [144]. This could indicate that ACPA responses to citrullinated antigens are non-overlapping and coexist in some individuals. Other studies suggest that these antibody responses at least are partially cross-reactive in a subpopulation of RA individuals [144,146].

**Figure 5:**
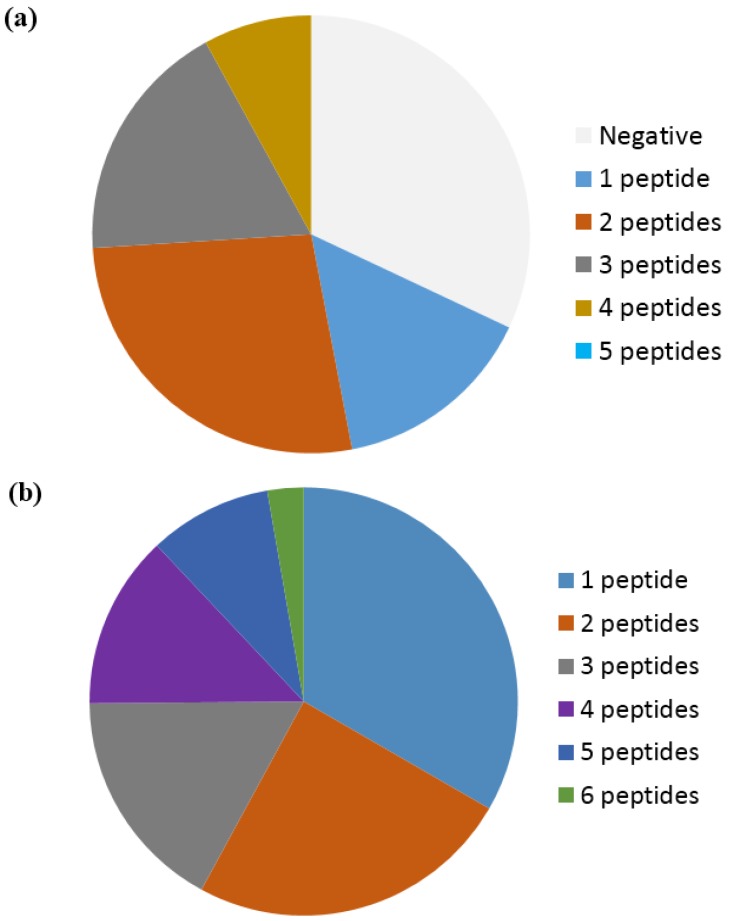
Reactivity of rheumatoid arthritis sera to linear citrullinated peptides. (**a**) Reactivity of 60 RA sera to five selected EBNA-1 peptides [136]; (**b**) Reactivity of 141 RA sera to six citrullinated peptides (vim^60−75^, α-enolase^15−21^, fibrin^617−31^, col-II^358−75^, filaggrin^306−24^, PAS^621−40^) [113].

Based on a study by Ioan-Facsinay et al. analyzing ACPA reactivity to selected citrullinated peptides, it was concluded that ACPAs are a collection of antibodies that are divided into overlapping and non-overlapping reactivities [143]. Moreover, it was confirmed that the majority of ACPA responses are cross-reactive; however, this cross-reactivity is not complete, as distinct non-cross-reactive responses occasionally are detected in RA patients [143], which is nicely illustrated in Figure 4. These findings are in accordance to the fact that cross-reactive antibodies, recognizing multiple epitopes, primarily are dependent on a central Cit motif in combination with a random peptide backbone, as previously described [135]. To study whether non-cross-reactive ACPAs can exist within an individual, we recently determined the cross-reactivity of RA sera [80,116]. Using different citrullinated peptides, we found that the degree of cross-reactivity varied and, more importantly, that non-overlapping ACPA responses exist. As depicted in Figure 5a, illustrating the reactivity of RA sera to various citrullinated peptides, 15% of the RA sera were monospecific to the respective citrullinated EBNA-1 peptides (sera reacting with only one citrullinated peptide) [79]. Furthermore, although a small portion of the RA sera was monospecific, 50% of the RA sera reacted with more than two citrullinated EBNA-1 peptides, confirming the theory of overlapping and non-overlapping ACPA reactivities [47,79]. Similar findings have been repeated with other citrullinated peptides, e.g., citrullinated fibrinogen, α-enolase and collagen, where both cross-reactive and monospecific ACPA responses were identified, as illustrated in Figure 5b [115]. Moreover, it has been verified that patients with RA and various reactivities against one or more citrullinated peptides do not present any difference in their clinical picture or in disease severity [147]. The conclusions are based on reactions of polyclonal patient sera and remain to be verified with larger collections of monoclonal antibodies (which are not available at present). The monoclonal antibodies investigated to date show broad cross-reactivity, reflecting a high backbone dependency (in addition to the central Cit-Gly motif). However, the existence of more specific side chain–dependent antibodies is indicated by the reactivity pattern of patient sera (Figure 5).

When conducting further experiments, we confirmed that ACPA responses are a group of antibodies with varying degrees of cross-reactivity. Analyzing antibody reactivity to multiple citrullinated peptides, among others, fibronectin, fibrinogen and filaggrin showed that multiple citrullinated peptides were recognized [131,133]. Screening of these patient sera using Ala-substituted peptides indicated that these peptides primarily were dependent on a central Cit-Gly motif, apparently in combination with a random peptide backbone [135]. Nevertheless, a small subgroup of patient sera was less cross-reactive, only recognizing a single or very few citrullinated peptides. Screening of these patient sera using alanine-substituted peptides indicated that the majority of the amino acids in the 14-mer pro-filaggrin peptide were essential for antibody reactivity (unpublished data). Thus, in this case, an actual epitope-specific ACPA response was identified, which is in great contrast to other studies indicating that ACPA reactivity is primarily dependent on the presence of a Cit-Gly motif [79,131]. Based on these results, we emphasize that “overlapping” cross-reactive ACPA reactivities primarily depend on a central Cit-Gly motif in combination with a flexible peptide backbone, whereas the “non-overlapping” non-cross-reactive ACPA reactivities depend on a citrullinated epitope, primarily being side chain–specific. However, this remains to be verified.

The polyclonal nature of ACPA responses in RA patients complicates the study of antibody characteristics such as cross-reactivity or affinity. What complicates the discussion about ACPAs even further is that in contrast to an antibody response to a specific protein, ACPAs are in fact a group of antibodies, each with its own citrullinated protein target. One way to circumvent these issues is to analyze the ACPA responses at a clonal level. Studies by us and van de Stadt et al. tested the reactivity of monoclonal antibodies to various citrullinated epitopes [80,133]. Analyzing the reactivity of these antibodies, significant reactivity was found to the target citrullinated protein, but distinct cross-reactivity was found towards citrullinated peptides derived from other proteins as well [80,135]. Moreover, findings by van de Stadt showed that the antibodies were derived from different naïve B cells that underwent extensive somatic hypermutation [80]. These results were further supported by recent studies, in which monoclonal antibodies generated against citrullinated collagen and fibrinogen, respectively, cross-reacted with other citrullinated antigens [44,133]. These findings are in accordance with studies performed by Ioan-Facsinay et al., where affinity-purified antibodies were able to recognize several citrullinated targets [143]. Thus, monoclonal and polyclonal antibody responses are similar in relation to ACPA reactivity, indicating that the identified ACPA reactivity pattern in human sera is the result of the presence of (partially) cross-reactive antibodies and not just the presence of co-existing mono-specificities.

## 5. Conclusions

The fact that ACPAs recognize multiple targets primarily containing essential Cit-Gly motifs has resulted in several studies analyzing ACPA reactivity to several citrullinated targets, as described in this review.

Collectively, ACPAs are a large collection of well-characterized, largely cross-reactive, but occasionally non-cross-reactive, fine specificities. However, the crucial importance of ACPA as a diagnostic marker and a potential contributor to the pathogenesis of RA has left limited room for thorough characterization of the actual antibody-antigen interface between ACPAs and their citrullinated targets. It is well described and accepted that ACPA reactivities primarily are of a cross-reactive nature; however, only limited knowledge is available in relation to the properties of the non-cross-reactive ACPAs. Studies indicate that the cross-reactive and non-cross-reactive ACPA responses in theory are two separate groups of antibodies. Other studies find that the cross-reactive ACPAs primarily depend on a central Cit-Gly motif in combination with the peptide backbone for reactivity. We suggest that non-cross-reactive ACPAs depend on a specific citrullinated epitope, rather than a citrullinated motif, thus making the fine specificities of the two groups of ACPAs different from each other. Focusing on the characterization of the non-cross-reactive antibody responses may reveal the true autoantigens of RA and contribute to understanding their nature, origin and potential function in the onset of RA.

## Figures and Tables

**Table 1:** Physiological functions of citrullination mediated by peptidylarginine deiminases.

Physiological Functions of Citrullination	References
Skin keratinization	[7]
Brain development	[7,21]
Fertility	[20]
Hair formation	[22]
NET formation	[37]
Gene regulation	[24,25,26,27,28,29,30]
Immune functions	[7,33,34,35,36]

NET, neutrophil extracellular trap.

**Table 2:** Specificity and sensitivity of anti-citrullinated protein antibodies.

Antibody	Specificity	Sensitivity	Reference
Anti-perinuclear factor	90	50–70	[68]
Anti-keratin antibodies	94	45	[69]
Anti-citrullinated filaggrin	>90	60	[70]
Anti-citrullinated fibrinogen	>90	55	[71]
Anti-fibrin	90	75	[72]
Anti-CCP1	96	53	[63]
Anti-CCP2	94–95	68–74	[73,74]
Anti-CCP3	86–96	68–79	[67,73,74,75]
Anti-CCP3.1	98	83	[74]
Anti-Sa antibodies	99	43	[76]
Anti-mutated citrullinated vimentin	97	72	[77]
Anti-citrullinated EBNA-1	85	67	[78,79]

CCP, cyclic citrullinated peptide; EBNA, Epstein-Barr nuclear antigen; Sa, Savoie.

**Table 3:** Reactive anti-citrullinated protein antibody epitopes.

Protein	Epitope	No of Cit Residues	Most Important Residue	Reference
Enolase	^5^ KIHA-Cit-EFDS-Cit-GNPTVE ^21^	2	Cit ^15^	[121]
Fibrin	α^36^ GP-Cit-VVE-Cit-HQSACKDS ^50^	2	Cit ^42^	[73]
Fibrin	β^60^ Cit-PAPPISGGGY-Cit-A-Cit ^74^	3	Cit ^74^	[73]
Collagen II	^358^ GA-Cit-GLTG-Cit-PGDAGPPGPP ^375^	2	Cit ^360^	[44]
Filaggrin	^306^ SHQEST-Cit-G-Cit-SRGRSGRSG ^324^	2	Cit ^312^	[3]
EBNA-1	GGRRGRGRERA-Cit-GGSRERAR	1	-	[79]
EBNA-1	ARGGSRERARGRGRG-Cit-GEKR	1	-	[79]

EBNA, Epstein-Barr nuclear antigen.
